# Evidence for genetic differentiation at the microgeographic scale in *Phlebotomus papatasi* populations from Sudan

**DOI:** 10.1186/1756-3305-5-249

**Published:** 2012-11-12

**Authors:** Noteila M Khalid, Marium A Aboud, Fathi M Alrabba, Dia-Eldin A Elnaiem, Frederic Tripet

**Affiliations:** 1 Department of Zoology, Khartoum College of Medical Science, PO Box 10995, Khartoum, Sudan; 2 Department of Biological Sciences, Faculty of Science, Al-Neelain University, Khartoum, Sudan; 3 Department of Zoology, Faculty of Science, University of Khartoum, PO Box 321, Khartoum, Sudan; 4 Department of Natural Sciences, University of Maryland Eastern Shore, Princess Anne, MD, USA; 5 Centre for Applied Entomology and Parasitology, Keele University, Keele, Staffordshire, ST5 5BG, UK

**Keywords:** *Phlebotomus papatasi*, Sudan, Gene flow, Genetic differentiation

## Abstract

**Background:**

Cutaneous Leishmaniasis (CL) is endemic in Sudan. It is caused by *Leishmania major* parasites and transmitted by *Phlebotomus papatasi* sandflies. Recently, uncommon clinical manifestations of CL have been reported. Moreover, *L*. *donovani* parasites that cause Visceral Leishmaniasis (VL) have been isolated from CL lesions of some patients who contracted the disease in Khartoum State, Central Sudan with no history of travelling to VL endemic sites on south-eastern Sudan. Because different clinical manifestations and the parasite behaviour could be related to genetic differentiation, or even sub-structuring within sandfly vector populations, a population genetic study was conducted on *P*. *papatasi* populations collected from different localities in Khartoum State known for their uncommon CL cases and characterized by contrasting environmental conditions.

**Methods:**

A set of seven microsatellite loci was used to investigate the population structure of *P*. *papatasi* samples collected from different localities in Khartoum State, Central Sudan. Populations from Kassala State, Eastern Sudan and Egypt were also included in the analyses as outgroups. The level of genetic diversity and genetic differentiation among natural populations of *P. papatasi* was determined using *F*_*ST*_ statistics and Bayesian assignments.

**Results:**

Genetic analyses revealed significant genetic differentiation (*F*_ST_) between the Sudanese and the Egyptian populations. Within the Sudanese *P. papatasi* populations, one population from Gerif West, Khartoum State, exhibited significant genetic differentiation from all other populations including those collected as near as 22 km.

**Conclusion:**

The significant genetic differentiation of Gerif West *P*. *papatasi* population from other Sudanese populations may have important implication for the epidemiology of leishmaniasis in Khartoum State and needs to be further investigated. Primarily, it could be linked to the unique location of Gerif West which is confined by the River Nile and its tributaries that may act as a natural barrier for gene flow between this site and the other rural sites. The observed high migration rates and lack of genetic differentiation among the other *P*. *papatasi* populations could be attributed to the continuous human and cattle movement between these localities.

## Background

Female sandflies of the genus *Phlebotomus* in the Old World are vectors of *Leishmania* parasites, the causative agent of different clinical forms of leishmaniasis [1]. The disease is endemic in 88 countries in five continents with a total of 350 million people at risk [2].

The parasites within the *Leishmania donovani* complex usually invade the macrophages of the liver, spleen and bone marrow causing the severe symptoms of the fatal Visceral Leishmaniasis (VL) [3,4]; while the parasites of *L*. *major* complex which cause Cutaneous Leishmaniasis (CL) invade the subcutaneous reticulo-endothelial system and cause self-healing lesions that leave life-long scars [5,6].

*Phlebotomus papatasi* (Scopoli, 1786) is the principal vector of *Leishmania major* in the Old World [1]. It is a widely distributed species found in variable habitats and associated with a wide range of vertebrate hosts [7,8]. Due to their poor dispersal capacity, populations of *P*. *papatasi* are expected to show some genetic structuring along their geographical range as a result of adaptation to local habitats and limited gene flow [9]. Such genetic variability could play an important role in the epidemiology and clinical manifestations of leishmaniasis since it affects the vectorial capacity of the vector [10,11].

Previous studies focusing on potential population sub-structuring in *P*. *papatasi* used a number of molecular markers and provided contrasting results. For example, isoenzyme analyses clearly separated Western Mediterranean *P*. *papatasi* populations from those from eastern countries but, predictably, failed to reveal differences between urban and rural sandfly populations in Morocco [12]. Mitochondrial DNA sequences coding for *cytochrome b* (mtDNA *cyt b*) and sequences from the second internal transcribed spacer of the ribosomal DNA (rDNA ITS2) were both used to study the population structure of *P*. *papatasi* populations from North Africa and the Mediterranean sub-region countries [13,14]. The analyses of mtDNA *cyt b* sequences suggested some level of genetic differentiation among widely separated populations and revealed a pattern of isolation-by distance between populations from Syria, Egypt, Israel/Palestine and Turkey [13]. The ND4 mtDNA region was also used in combination with the rDNA ITS2 to study the population structure of *P*. *papatasi* from 18 countries from North Africa, the Mediterranean sub-region, Saudi Arabia and India, but revealed no clear phylogeographic structure between those populations. However, signs of restricted gene flow were found among populations from Iran, Egypt, Syria, Yemen and Turkey [14].

Microsatellite markers combined with Bayesian statistic analysis were recently used to study the population structure of *Phlebotomus papatasi* populations in countries from the North-African and the Mediterranean sub-regions. This study confirmed the occurrence of highly significant genetic differentiation between some populations [15]. However, the geographical scale of the study did not allow for detecting possible genetic differentiation at the local level, which may be the most relevant for explaining the observed patterns of variation in epidemiologically relevant traits observed in some regions.

Thus, so far, genetic differentiation among *P*. *papatasi* populations could not be demonstrated at the local geographical level, despite evidence suggesting that it may occur. For example, Schmidt and Schmidt (1963) observed marked morphometric variations within the Egyptian populations of *P*. *papatasi* suggestive of sub-structuring [16]. Using isoenzyme analysis, Kassem *et al*. reported the presence of polymorphisms at many isoenzyme loci among populations of *P*. *papatasi* from Egypt but no significant genetic differentiation could be detected [17,18]. It must be stressed that the possibility of genetic differentiation in *P*. *papatasi* populations at the local scale has not yet been explored using microsatellite markers which, with their higher mutation rates, should be comparatively much more informative than isoenzymes and other sequence loci [19].

In Sudan, CL is caused by *L*. *major* and transmitted by *P*. *papatasi*[20,21]. Before the 1970s, the disease was confined to the western parts of the country. Thereafter, major epidemics occurred along the River Nile and the disease became endemic in many regions of the country [21]. The usual clinical forms of the disease usually heal spontaneously without the need for medical treatment, a matter which discourages patients from attending the health centres, and therefore obscures the incidence of the disease [22]. Visceral Leishmaniasis (VL) which is caused by *L*. *donovani* and transmitted by *P*. *orientalis* is known to be endemic in eastern and southern parts of Sudan [4,23], with few scattered foci along the White Nile and Darfur [24]. Recently, uncommon clinical manifestations of CL that did not heal spontaneously nor responded to usual drugs have been reported [25]. Moreover, *L*. *donovani* parasites have been isolated from CL lesions of some patients who contracted the disease in Khartoum State, Central Sudan, with no history of travelling to VL endemic sites [26]. Recent studies have demonstrated the possibility of genetic exchanges between different strains and species of the *Leishmania* parasites [27-29] which may further complicate the epidemiology of the disease since hybrid parasites may adapt differently to the vector and reservoir hosts [30].

Because different clinical manifestations and the parasite behaviour may also be related to genetic differentiation or sub-structuring within sandfly vector populations [11,31], we conducted a population genetic study of *P. papatasi* populations in Sudan from broad to local geographical scale. This was done using a set of 5 microsatellite markers, especially developed for *P*. *papatasi*[32], and given the paucity of such markers, by testing and using additional markers developed for *P*. *perniciosus* a related phlebotomine species [33].

*P*. *papatasi* populations were collected from different localities characterized by the distribution of the atypical CL cases. The level of genetic diversity and genetic differentiation among natural populations of *P. papatasi* was determined using *F*_*ST*_ and Bayesian assignments. Identifying potential factors leading to genetic differentiation and structuring in *P*. *papatasi* populations might improve our understanding of the epidemiology of the disease and help develop appropriate control strategies.

## Methods

### Study sites

**Figure 1:**
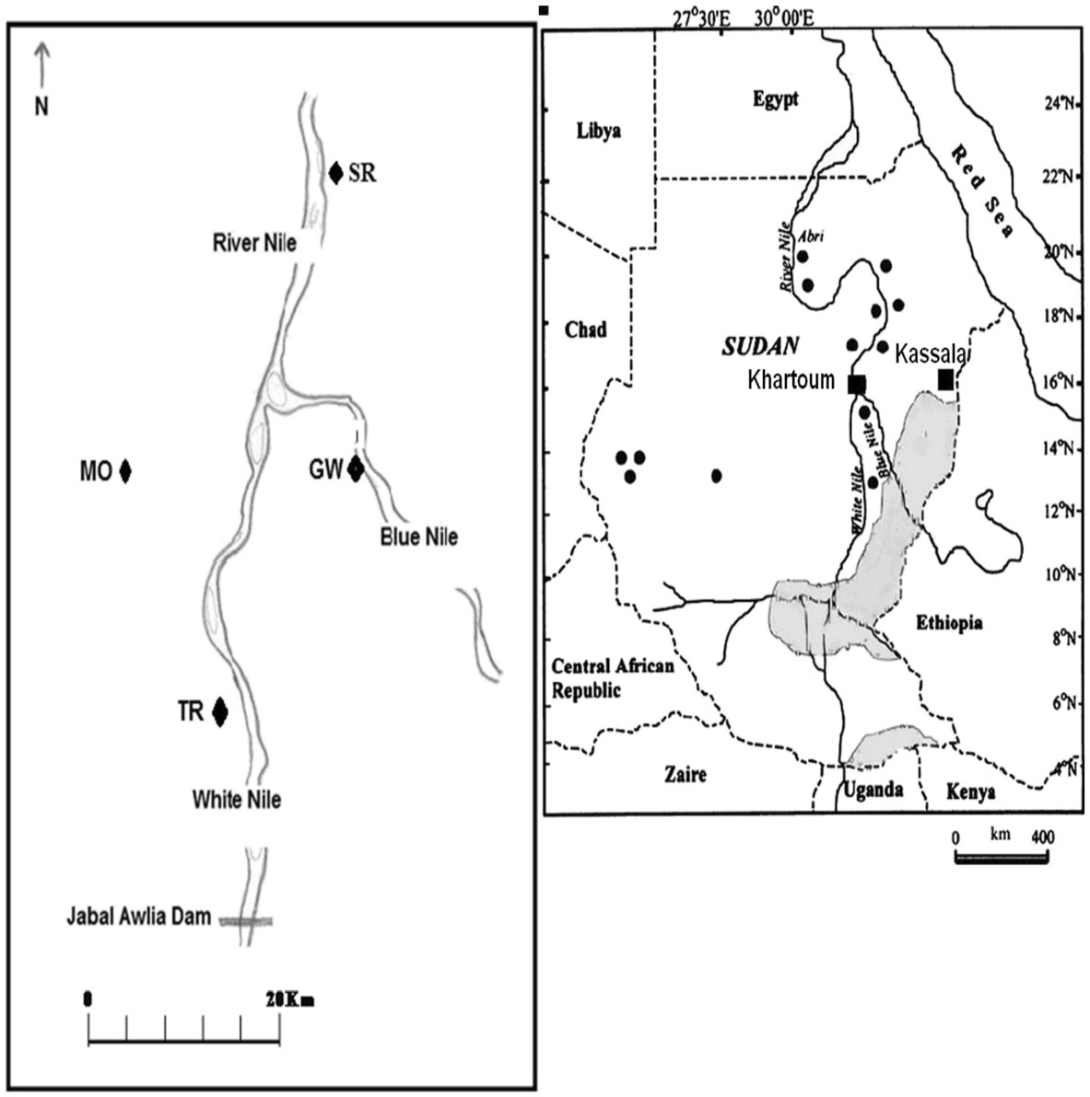
**Map of the Sudan showing leishmaniasis endemic sites and the main study sites from which *Phlebotomus papatasi *populations were collected.** VL endemic area (grey area), CL endemic sites (●), Khartoum and Kassala States (■) (right). Map of the main Study sites in Khartoum State (left).

A total of 126 male specimens of *P. papatasi,* which are typically easier to identify morphologically than females in this and other phlebotomine species, were collected from five different localities in Sudan separated by 20 to 440 Km (Figure 1, Table 1). Individuals from a colonized population from Egypt (EGY) (~1600 Km from Khartoum State) were also included. The Sudan study sites were selected according to the distribution of the uncommon CL cases. They also represent different ecological zones separated by the River Nile and its tributaries. Four sites were located in Khartoum State; namely, Moyleih (MO), a semi-desert area on the west bank of the White Nile, El-Trais (TR), an irrigated area on the west bank of the White Nile; Sirougia village (SR) is a green rich irrigated area with many horticultural schemes on the east bank of the River Nile, northern Khartoum and Gerif West (GW) used to be green farms on the west bank of the Blue Nile, but recently changed into a residential area (also known as El-Manshya). Cutaneous leishmaniasis is not common in Moyleih and El-Trais areas; however some of the patients with CL lesions due to *L*. *donovani* were from these sites (Pers. Comm. Prof M. M. Mukhtar, Institute of Endemic Diseases, Sudan).Sirougia is a well-known endemic focus of CL where previous epidemic occurred beside some of the uncommon CL cases came from the nearby villages (Pers. Comm. Prof A. M. Musa, Institute of Endemic Diseases, Sudan). Gerif West is not known to be endemic for cutaneous leishmaniasis. However, some of the uncommon CL and CL due to *L*. *donovani* cases were from this site. The fifth Sudan study site was the village of Wad Shariefai (SHR) in Kassala State, Eastern Sudan which is located in the immediate vicinity of the dry savannah habitats of the Tajouj Forest. Wad Shariefai village is a site where three closely-related species belonging to the subgenus *Phlebotomus phlebotomus* (*P*. *papatasi*, *bergeroti* and *duboscqi*) were found sympatrically. Due to the far location of this Eastern site it is expected that *P*. *papatasi* from this site would be genetically different from *P*. *papatasi* populations from Khartoum Sate, Central Sudan.

**Table 1:** **Origin and number of specimens (*N*) used in a population genetic study of *Phlebotomus papatasi *collected from Sudan and Egypt**

**Location**	**Latitude**	**Longitude**	***N***	Disease endemicity *
Moyleih, MO	15° 34’ 644” N	32° 23’ 160” E	26 males	Scattered CL cases
El-Trais, TR	15° 23’ 003” N	32° 26’ 970”E	24 males	Scattered CL cases
Gerif west, GW	15° 35’ 394” N	32° 35‘ 160” E	25 males	Uncommon CL cases
Sirougia, SR	15° 47‘ 509” N	32° 33’ 471” E	25 males	Uncommon CL cases
Wad Shariefai, SHR	15° 22’ 49” N	36° 25’ 98” E	26 male	Rare CL cases
Colony, EGY†	30^ο^ 03’ --“ N	31^ο^ 13’ --“E	25 males	

*According to the personal communications and the reported cases of leishmaniasis by the National Leishmania Control Program, Federal Ministry of Health, Sudan (2008 to 2010) either from the same study site or the nearest village to it.

† The exact collection site of the colony is unknown.

### Microsatellite PCR amplification

Samples were processed and DNA was extracted as previously described [34]. Microsatellite loci were amplified using the PCR primers originally developed by Hamarsheh *et al*. and Aransay *et al*. [32,33]. The PCR reactions were performed in a gradient thermocycler (PT200, MJ) for 30 cycles. The PCR mix contained 1.5 to 3μl of genomic DNA (5–8ng/μl) extracted from individual male sandfly, 5x PCR buffer (Promega, Madison, WI), 15pmol of each primer, 200mM of each dNTPs, 1.2μl of 25mm MgCl_2_ and 0.5U Taq ® DNA polymerase (Promega, Madison, WI) in 20μL total reaction volume. The thermal cycling conditions were an initial hold at 95°C for 2min, followed by 30 cycles of 94°C for 30s, 55°C for 30s and 72°C for 30s and a final extension at 72°C for 5min. For amplifications involving the primer *AA13*, a touchdown PCR programme was performed. The programme steps were as mentioned above except that the number of cycles was increased to 35 and the starting annealing temperature was 59°C, which was then decreased by 1°C for the first five cycles. For correct sizing of polymorphic PCR products, the forward primers were labelled with 5’-fluorescent tags. Details of the primers sequences; repeat motif, annealing temperature, and fluorescent labelling are given in Table 2.

**Table 2:** **Repeat motif, primers sequence, and annealing temperature used for PCR amplification of the microsatellite loci in *Phlebotomus papatasi *collected from different populations in Sudan and Egypt**

**Locus**	**Motif**	**Fluorescent labeled primer sequence**^**†**^	**Annealing T°**
*Pap1	(GTT)	**FAM**-CGGTCACTTCCCTCTTCTCA	58°C
		R: CCCCTTCTACAACCACTTCAA	
*Pap2	(CA)	**JOE**-CACTCGCAAGATGGTAGGGTA	58°C
		R: CGGCGCCTATAGACAGAGAA	
*Pap4	(GAA)	**FAM**-GTGGCAAAATTGGTTTGGT	55°C
		R: ACCTTTTGCATATGCCCATC	
^*♦*^*AA13*	(GAG)	**TAMRA**-CTCCAACTCCTCATCCACCTC	59/55°C
		R: GGCGACGAATGGGACAAAG	
^♦^*AA18*	(AGC)	**JOE**-5’-GCTGCACAGCCGCCTGATG	55°C
		R: ATCAGCAGACACTCCAGCAACACC	
^♦^*AA24*	(GCA)	**TAMRA**-5’-CTATTCCCGCCCCACTTGG	55°C
		R: TCAATCGACATTCGGACAGGC	
^♦^*AA82*	(GGA)	**JOE**-TCAACCAAGGTAGTCTCATCAG	49°C
		R: GAGGACTTCCGATTTCTGTAG	

† Fluorescent marker dyes used in this study are indicated in bold.

*Designed by Hamarsheh *et al*. (2006) for *P*. *papatasi*.

^♦^Designed by Aransay (2001) for *P*. *perniciosus*.

### PCR product multiplexing

PCR product multiplexing was done according to the amplified allele size and the fluorescent dye labelling of each product in order to create two primer sets. Set A contained a mix of *Pap1*, *Pap2*, *AA13*, and *AA82*, while set B was a mix of *Pap4*, *AA18*, and *AA24*. About 16 samples with different dilutions (1:10, 1:20, and 1:50) were initially genotyped at Eurofins MWG Operon Company, Ebersberg, Germany. According to the results from the first genotyped samples, a suitable dilution factor was chosen and the two sets of sample microplates were prepared, tightly packaged in dry ice and submitted for subsequent genotyping in the aforementioned company.

### Microsatellite data analysis

Because the repeat arrays of all the microsatellite loci consist of trinucleotide repeats (except for *Pap2* which consist of dinucleotide repeats), the allele sizes were expected to differ predominantly by multiples of three nucleotides. Therefore any exceptions were treated as a result of insertions or deletions (indels) of odd numbers of bases in the flanking region.

The data were screened for the presence of stutters, null alleles and large allele dropout using the software Microchecker version 2.2.3 available at http://www.microchecker.hull.ac.uk[35]. Then the data were analyzed using ARLEQUIN version 3.5 available at http://cmpg.unibe.ch/software/arlequin3[36]. The programme was used to calculate allele frequencies at each microsatellite locus and to test whether they conformed to Hardy-Weinberg equilibrium (HWE) in each population. The same programme was used to estimate genetic differentiation (*F*_ST_) between populations and the level of gene flow (*Nm*) among them, as well as assessing whether patterns of genetic divergence among *P*. *papatasi* populations followed an isolation-by-distance pattern. The genotype assignment test implemented in ARLEQUIN programme was used to assign individual sandfly genotypes to their population of origin by computing the log-likelihood of each individual multi-locus genotype in each population sample, assuming that the individual belongs to that population [36]. The significance of all tests (*P* <0.05) was obtained with 1000 permutations and 100000 Markov chain steps.

Hidden population sub-structure within *P*. *papatasi* populations were further investigated using the Bayesian clustering algorithm implemented in the software STRUCTURE version 2, available at http://pritch.bsd.uchicago.edu[37]. The programme was run using an admixture model with a burn-in period of 10000 iterations, followed by 100000 iterations of Markov chain Monte Carlo (MCMC) repeats for each setting of K from 1 to 10 and no prior information about the population structure was provided. After calculations, the value of the ad hoc ΔK was plotted against the assumed number of populations (K = 10), the highest peak of the graph was considered as the number of possible subpopulations in the data set.

## Results

### Allelic richness and heterozygosity

The three markers *Pap1*, *Pap2* and *Pap4* showed varying degrees of heterozygosity among the different study populations; although the two markers *Pap3* and *Pap5* did not work well with the Sudanese *P*. *papatasi* populations even after several trials with different amplification conditions. Amongst the markers for *P*. *perniciosus*, *AA13*, *AA18*, *AA24* and *AA82*, were successfully amplified in *P. papatasi*. However, these markers tended to be less polymorphic than in the original species. The total number of alleles per locus ranged from 1 to 5 with locus *AA18* being monomorphic in all populations. In addition, each of the two loci *AA13* and *AA24* were found to amplify two different polymorphic loci with distinct allele size ranges (i.e. *AA13a*: 81-96bp and *AA13b* 129-132bp; *AA24a*: 141–147 and *AA24b*: 255–273) which were not found to be in linkage disequilibrium. This suggests that these two loci were the results of duplications to different unlinked regions of the genome; thus they were treated as two additional loci thereby increasing the total number of loci examined to nine (Table 3).

**Table 3:** **Number of alleles, expected mean heterozygosity (He) and allelic range per microsatellite locus within *Phlebotomus papatasi *individuals collected from different populations in Sudan and Egypt**

**Locus**	**No. of alleles (*k*) and sample sizes (*n*)**						**He ± SD**	**Allelic range (bp)**
	**MO**	**TR**	**GW**	**SR**	**SHR**	**EGY**		
*Pap1*	5(26)	2(24)	3(25)	3(25)	3(25)	3(25)	0.543 ± 0.06	125-143
*Pap2*	2(25)	2(24)	4(22)	4(24)	4(25)	5(25)	0.541 ± 0.17	83-91
*Pap4*	4(26)	4(24)	2(25)	4(25)	2(26)	1(25)	0.115 ± 0.09	87-96
*AA13a*	3(24)	2(23)	1(23)	3(24)	2(26)	1(25)	0.047 ± 0.05	81-96
*AA13b*	2(25)	2(24)	2(23)	2(23)	2(25)	2(25)	0.495 ± 0.01	129-132
*AA24a*	2(26)	2(24)	2(25)	3(25)	2(26)	2(22)	0.508 ± 0.07	141-147
*AA24b*	4(26)	5(24)	4(22)	5(24)	5(24)	4(23)	0.505 ± 0.06	255-273
*AA82*	2(20)	2(18)	2(22)	2(9)	2(15)	2(21)	0.092 ± 0.07	141-159
*AA18*	1(26)	1(24)	1(23)	1(25)	1(25)	1(25)	-	111

SD = Standard deviation.

There was no evidence for scoring error due to stuttering, large allele dropout or null alleles after checking the dataset with Microchecker software. Except for locus *AA18* which was monomorphic in all *P*. *papatasi* populations, the other eight studied microsatellite loci were found to be polymorphic. The total number of alleles per locus ranged from 2 to 5 with an average of 2.77 alleles per locus and an average gene diversity (mean expected heterozygosity) of 0.355 ± 0.226 (Table 3).

The six *P*. *papatasi* populations were found to be polymorphic at 88.9% of the amplified loci. Allele frequency distribution between the 6 *P*. *papatasi* populations varied from locus to locus, with some loci having similar distributions and others being highly variable. For example, allele 128 for locus *Pap1* was the most common allele among all Sudanese populations while allele 125 was the most common among the Egyptian population. For locus *Pap2*, allele 85 was the most common among populations from Sudan while allele 89 was the most common in a population from Egypt. Private alleles were detected at locus *Pap1* in the MO population, locus *AA24a* in the SR population, loci *AA13a* and *AA24b* in the SHR population, and locus *Pap2* in population EGY. Locus *AA18* was monomorphic and was excluded from subsequent analyses (Table 4).

**Table 4:** **Allelic frequencies for polymorphic microsatellite loci in individuals of *Phlebotomus papatasi *collected from different populations in Sudan and Egypt**

**Locus**	**Allele size (bp)**	**MO**	**TR**	**GW**	**SR**	**SHR**	**EGY**
*Pap1*	125	0.404	0.438	0.360	0.260	0.380	0.520
	**128**	0.539	0.563	0.620	0.700	0.580	0.300
	131	0.0192	0.000	0.020	0.000	0.040	0.180
	134	0.0192	0.000	0.000	0.040	0.000	0.000
	143	0.0192*	0.000	0.000	0.000	0.000	0.000
*Pap2*	83	0.000	0.000	0.000	0.000	0.000	0.020*
	**85**	0.720	0.896	0.795	0.625	0.700	0.180
	87	0.280	0.104	0.045	0.104	0.100	0.020
	89	0.000	0.000	0.023	0.083	0.100	0.480
	91	0.000	0.000	0.136	0.188	0.100	0.300
*Pap4*	87	0.019	0.021	0.000	0.040	0.000	0.000
	90	0.038	0.021	0.040	0.040	0.019	0.000
	**93**	0.885	0.917	0.960	0.900	0.981	1.000**†**
	96	0.058	0.042	0.000	0.020	0.000	0.000
*AA13a*	81	0.042	0.022	0.000	0.021	0.000	0.000
	87	0.000	0.000	0.000	0.000	0.019*	0.000
	90	0.021	0.000	0.000	0.021	0.000	0.000
	**96**	0.938	0.978	1.000**†**	0.958	0.981	1.000**†**
*AA13b*	**129**	0.560	0.563	0.630	0.544	0.520	0.540
	132	0.440	0.438	0.370	0.457	0.480	0.460
*AA82*	141	0.000	0.000	0.0227*	0.000	0.000	0.000
	**150**	0.975	0.972	0.977	0.889	0.933	0.930
	159	0.025	0.028	0.000	0.111	0.067	0.070
*AA24a*	141	0.000	0.000	0.000	0.040*	0.000	0.000
	144	0.423	0.438	0.800	0.340	0.539	0.410
	**147**	0.577	0.563	0.200	0.620	0.462	0.590
*AA24b*	255	0.000	0.000	0.000	0.000	0.021*	0.000
	258	0.135	0.063	0.182	0.167	0.208	0.020
	261	0.077	0.042	0.114	0.042	0.021	0.170
	**264**	0.692	0.771	0.614	0.688	0.667	0.650
	270	0.096	0.083	0.091	0.083	0.083	0.150
	273	0.000	0.042	0.000	0.021	0.000	0.000

Bold numbers indicate the most common allele.

* Private alleles.

**†** Monomorphic loci.

There was a considerable variation in mean heterozygosity in *P*. *papatasi* populations, including the Egyptian laboratory colony, despite its long history of colonization. Surprisingly, this population was the most variable with approximately 0.493 heterozygosity (i.e. on average 49% of the individuals were heterozygous at any given locus). Meanwhile, MO population was the most heterogeneous among all the Sudanese populations (40% heterozygosity). The most uniform population in the study was TR with 31.5% heterozygosity (Table 5).

**Table 5:** **Number of alleles, average observed (Ho) and expected Heterozygosity (He) and allelic range for Sudanese and Egyptian populations of *Phlebotomus papatasi***

**Mean**	**MO**	**TR**	**GW**	**SR**	**SHR**	**EGY**
No. of alleles	3.00	2.50	2.67	3.33	3.00	2.83
Ho	0.404	0.315	0.361	0.350	0.376	0.493
He	0.356	0.283	0.306	0.387	0.362	0.407
Allelic range	9.833	7.833	7.200	10.000	8.000	7.600

### Linkage disequilibrium and Hardy–Weinberg equilibrium

As few as 14 out of 168 comparisons between pairs of loci were found to be in significant linkage disequilibrium. However, none of those comparisons remained significant after Bonferroni correction.

Each locus was tested for significant departure from Hardy-Weinberg equilibrium (HWE). Observed heterozygosity varied from 0.000 to 0.960 while expected heterozygosity ranged from 0.039 to 0.660. The observed heterozygosity was higher than the expected value in 25 cases and lower than expected in 13 cases of the comparisons made between *P*. *papatasi* populations (Table 6). Significant deviations from HWE due to heterozygotes deficiency were observed in locus *Pap2* within the SR and EGY populations, locus *Pap4* within the MO, TR, SR, and GW populations, and locus *AA24a* within SR population. This could be due to population subdivision rather than the existence of null alleles since amplification was successful for all individuals. Significant deviations from HWE due to homozygote deficiency were found in locus *Pap1* within GW population and locus *AA13b* in all populations. The Egyptian *P*. *papatasi* population was monomorphic for two loci (*Pap4*, and *AA13a*) and the GW population was monomorphic for *AA13a* (Table 6). Because of their consistent deviation from HWE among all populations, the two loci *Pap4* and *AA13*b were excluded from subsequent analyses.

**Table 6:** **Observed (Ho) and expected heterozygosity (He), probability of deviation from HWE, and inbreeding coefficient (*F***_**IS**_**) in populations of *Phlebotomus papatasi *collected from different sites in Sudan and Egypt**

**Locus**	**MO**	**TR**	**GW**	**SR**	**SHR**	**EGY**
***Pap1*** Ho	0.692	0.458	0.760	0.520	0.600	0.800
He	0.557	0.503	0.528	0.450	0.496	0.620
*P*	0.350	0.698	0.035*	0.807	0.485	0.196
*F*_IS_	−0.250	0.090	−0.452	−0.160	−0.216	−0.299
***Pap2*** Ho	0.400	0.208	0.227	0.375	0.480	0.320
He	0.411	0.191	0.354	0.568	0.450	0.660
*P*	1.000	1.000	0.087	0.026*	0.440	0.000*
*F*_IS_	0.028	−0.095	0.364	0.345	0.020	0.520
***Pap4*** Ho	0.154	0.083	0.000	0.120	0.039	Mono-
He	0.216	0.161	0.078	0.190	0.039	morphic
*P*	0.037*	0.042*	0.022*	0.045*	1.000	
*F*_IS_	0.293	0.486	1.000	0.374	−0.000	
***AA13a*** Ho	0.125	0.043	Mono-	0.083	0.039	Mono-
He	0.122	0.043	morphic	0.083	0.039	morphic
*P*	1.000	1.000		1.000	1.000	
*F*_IS_	−0.030	0.000		−0.011	0.000	
***AA13b*** Ho	0.880	0.875	0.739	0.913	0.960	0.920
He	0.503	0.503	0.476	0.507	0.509	0.507
*P*	0.000*	0.000*	0.008*	0.000*	0.000*	0.000*
*F*_IS_	−0.778	−0.769	−0.571	−0.833	−0.920	−0.846
***AA24a*** Ho	0.615	0.708	0.320	0.360	0.462	0.636
He	0.498	0.503	0.327	0.509	0.507	0.495
*P*	0.256	0.091	1.000	0.020*	0.705	0.215
*F*_IS_	−0.242	−0.422	0.020	0.296	0.091	−0.295
***AA24b*** Ho	0.538	0.417	0.455	0.542	0.542	0.565
He	0.497	0.400	0.582	0.501	0.515	0.532
*P*	0.684	0.674	0.109	0.879	1.000	0.255
*F*_IS_	−0.085	−0.043	0.224	−0.083	−0.053	−0.063
***AA82*** Ho	0.050	0.056	0.046	0.222	0.133	0.143
He	0.050	0.056	0.046	0.209	0.129	0.136
*P*	1.000	1.000	1.000	1.000	1.000	1.000
*F*_IS_	−0.000	0.000	0.000	−0.067	−0.037	−0.053

* Significant deviation from HWE (*P* < 0.05).

### Estimates of genetic differentiation between populations

**Figure 2:**
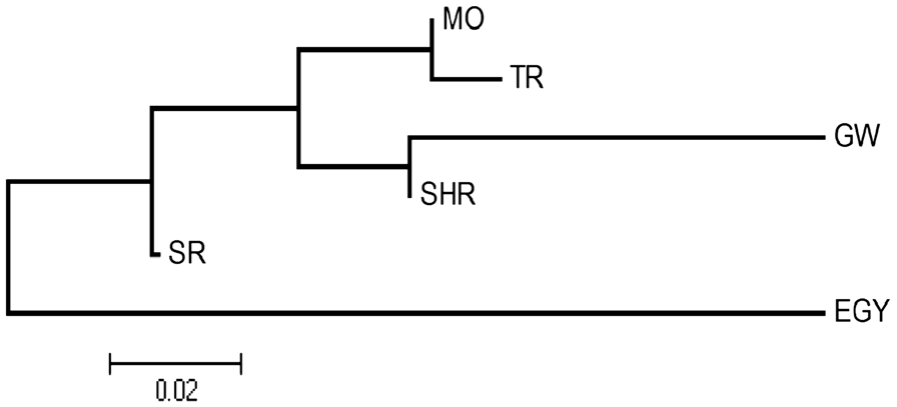
**Neighbour-joining (NJ) dendrogram showing the genetic distance between *Phlebotomus papatasi *populations from Sudan and Egypt.** Relationships based on Wright’s *F*_*ST*_ statistics

The genetic differentiation between pairs of populations, as measured by *F*_ST_ , ranged from 0.007 to 0.233. Significant *F*_ST_ estimates (*F*_ST_ =0.147-0.233) were observed between all *P*. *papatasi* populations from Sudan when compared to those from Egypt (Table 7). Significant genetic differentiation was also observed between *P*. *papatasi* populations from GW and SHR (*F*_ST_ =0.033), whereas moderate but non-significant differentiation (0.011-0.014) was observed between SHR and other populations from Khartoum State (MO, TR, and SR). Within Khartoum State, significant *F*_ST_ (0.100 to 0.125) estimates were also observed between GW and MO, TR, and SR populations. The populations from MO, TR, and SR showed little genetic differentiation amongst themselves as indicated by low *F*_ST_ values ranging from 0.007 to 0.046. A neighbour-joining tree based on all pair-wise *F*_ST_ estimates confirmed that the GW population, whilst being distinct from all other populations in Sudan is comparatively more similar to SHR than to nearer populations (Figure 2).

**Table 7:** **Estimates of gene flow *Nm *and geographical distances (km) (above diagonal), and genetic differentiation*F***_**ST **_**(below diagonal) between *Phlebotomus papatasi *populations from Sudan and Egypt**

	**MO**	**TR**	**GW**	**SR**	**SHR**	**EGY**
**MO**		72.33	4.38	20.49	44.79	2.33
		**(19.8)**	**(21.9)**	**(33.1)**	**(434.6)**	**(1614.7)**
**TR**	0.007		4.48	10.39	31.92	1.64
			**(27.6)**	**(47.5)**	**(426.7)**	**(1637.6)**
**GW**	0.102**	0.100**		3.49	14.53	1.65
				**(22.7)**	**(413.3)**	**(1615.3)**
**SR**	0.024	0.046*	0.125**		36.16	2.89
					**(416.51)**	**(1592.5)**
**SHR**	0.011	0.015	0.033*	0.014		2.88
						**(1718.3)**
**EGY**	0.177**	0.233**	0.232**	0.147**	0.148**	

The level of significance of *F*_ST_ estimates is indicated as * *P* < 0.05, ** P *<* 0.001.

As expected, *F*_ST_ estimates translated into high estimates of migration rate, *Nm*, among populations from MO and TR, Southwest Khartoum State. High gene flow estimates were also observed between populations from MO, TR and SR and SHR, despite the large geographical distance between them. However, very little gene flow was observed between those same populations and GW which is approximately geographically equidistant to the other three sites in Khartoum State (Table 7).

**Figure 3:**
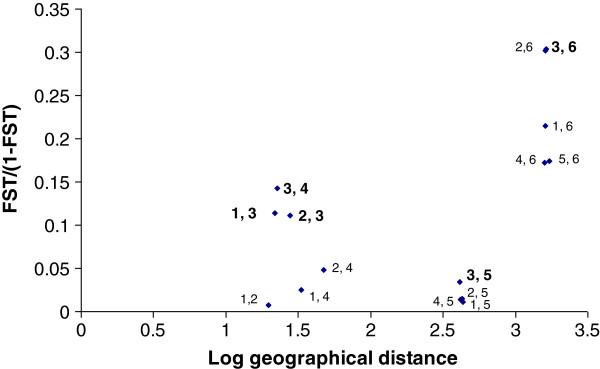
**Relationship between linearized *F***_**ST **_**and log geographic distance among *Phlebotomus papatasi *populations from Sudan and Egypt.** 1 = MO, 2 = TR, 3 = GW, 4 = SR, 5 = SHR, 6 = EGY (Mantel test: *P* > 0.05, non-significant).

A Mantel test showed that there was a positive but non-significant correlation (*r* = 0.08, *P* > 0.05) between linearized estimates of genetic differentiation *F*_ST_ (*F*_ST_/(1-*F*_ST_)) and geographical distances (log km) (Figure 3).

### Population structure

**Figure 4:**
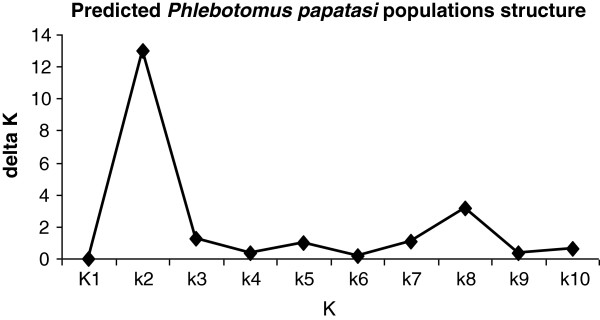
**Relationship between the second order rates of change of the likelihood function (Delta K) averaged over 10 runs (K).** The highest peak corresponds to the number of the possible subpopulations inferred by the software STRUCTURE (see text for details).

**Figure 5:**
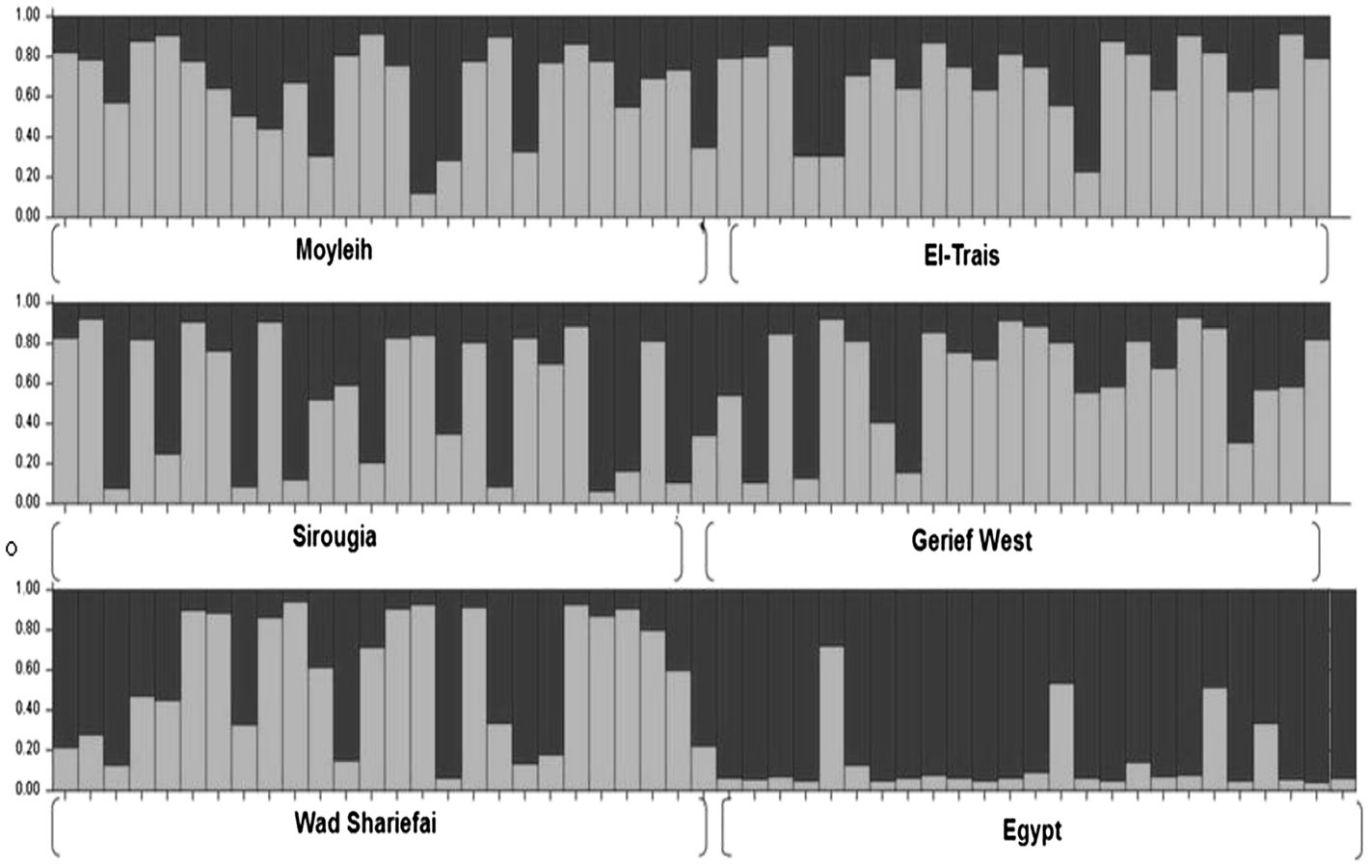
**Estimated membership coefficients of individual sandflies from six *P*. *papatasi *populations to the two putative populations predicted by program STRUCTURE.** Subpopulation (**A**) grey; subpopulation (**B**) black. Membership coefficients were estimated from microsatellite loci genotypic frequencies.

The STRUCTURE genotype assignment programme clustered our study populations into two groups (Figure 4). The dataset was further analyzed by sorting out individuals between the two suggested subpopulations as belonging to subpopulation A (grey colour) and subpopulation B (black colour) based on the likelihood of their genotype to belong to one or the other population (Figure 5). Most of the individuals in the Egyptian population (88%) were assigned to subpopulation B, while most of the individuals in the Sudanese populations belonged to subpopulation A. However, the observed heterogeneity in population assignment - e.g. 83% of TR and 73% of MO belong to the population A - suggested that the two clusters do not represent reproductively isolated populations (Table 8).

**Table 8:** **Assignment of different individuals of *Phlebotomus papatasi *sandflies collected from different locations in Sudan to two predicted subpopulations (A and B) using Bayesian assignments in STRUCTURE**

**Populations**	**MO**	**TR**	**SR**	**GW**	**SHR**	**EGY**
A^*^	73%	83%	52%	68%	50%	0
B^*^	23%	13%	40%	24%	46.2%	88%
**A/B**^**†**^	4%	4%	8%	8%	3.8%	12%
**TOTAL**	100%	100%	100%	100%	100%	100%

^*^Percentages are based on likelihood of each individual sandfly of belonging to subpopulations A and B.

^†^A/B = indicate the percentage of individuals which showed approximately equal probability of belonging to the A and B populations.

The same result was also observed when attempting to assign individual sandflies to the different collection sites by computing the log-likelihood of the genotype of each individual based on the allelic frequencies of each population. According to the population assignment test, about 80% of the Egyptian sandfly individuals were correctly assigned to their population of origin. However, none of the individual sandflies from the Sudanese populations of MO and TR were assigned to the Egyptian one (Table 9).

**Table 9:** **Number and percentage of individual *Phlebotomus papatasi *sandflies assigned to each collection site in Sudan and Egypt using the log-likelihoods approach in ARLEQUIN**

**Population**	**MO**	**TR**	**GW**	**SR**	**SHR**	**EGY**
**MO**	12 (**46%**)	6 **(25%)**	2 (8%)	1 **(4%)**	1 (4%)	1 (4%)
**TR**	7 **(27%)**	15 **(62.5%)**	4 **(16%)**	2 **(8%)**	5 **(19.2%)**	1 **(4%)**
**GW**	3 **(11.5)**	0	16 **(64%)**	2 **(8%)**	4 **(15.3%)**	1 **(4%)**
**SR**	3 **(11.5)**	2 **(8.3%)**	1 **(4%)**	17 **(68%)**	6 **(23%)**	1 **(4%)**
**SHR**	1 **(4%)**	1 **(4.2%)**	1 **(4%)**	1 **(4%)**	7 **(27%)**	1 **(4%)**
**EGY**	0	0	1 **(4%)**	2 **(8%)**	3 **(11.5)**	20 **(80%)**
**Total**	26	24	25	25	26	25

## Discussion

This study further validates the use of microsatellites for studying subtle patterns of genetic differentiation between populations at the microgeographic scale. In such situations, microsatellite markers with their high-mutation rates and high allelic diversity are known to perform much better than slow evolving allozymes and other nuclear and mitochondrial markers [38,39]. However, useful microsatellites are neither highly abundant nor easily isolated from arthropod species [40]. Homologous microsatellite loci have sometimes been amplified in species related to the one they were isolated from using the same primers, but amplification success strongly depends on the divergence time separating the species concerned [41].

Here, given the low number of microsatellite loci available for *P. papatasi,* additional loci originally designed for *P. perniciosus* were tested and successfully cross-amplified. As expected, some of these loci produced fewer alleles than in the original species (*P*. *perniciosus*). For example, the total number of alleles per locus for these loci ranged from 4 to 9 in 13 Spanish populations of *P*. *perniciosus* with a sample size ranging from 13 to 38 but here in our study allelic richness ranged from 1 to 5. Surprisingly, two primer pairs *AA24* and *AA13* amplified more than one locus in *P*. *papatasi* with distinct allele size-ranges and genetically unlinked, indicating a duplication of the genome in the region of those repeat sequences in *P. papatasi.* These loci were polymorphic in most *P. papatasi* populations, which contrasts with previous results in *P*. *perniciosus* where locus *AA24* was not significantly polymorphic in 12 out of 13 studied populations, and locus *AA13* was monomorphic in 5 of them [42].These variations suggest strikingly different evolutionary trajectories. Future comparative genomic studies might help elucidate the phylogenetic relationships and genome rearrangements characterizing these sandfly vector species.

Interestingly, no significant pattern of isolation-by-distance was observed amongst the 6 *P*. *papatasi* populations sampled from Central and Eastern Sudan, and Egypt. This result is due to the unexpected significant genetic differentiation found at the local geographical scale between the Sudanese populations that interfered with broader geographical patterns of genetic differentiation. Importantly, the *P*. *papatasi* population from GW was significantly genetically differentiated from that of SHR, located 400km away in Eastern Sudan. But the same population (GW) was also significantly genetically differentiated from the other 3 populations from Khartoum State (MO, TR and SR) despite these populations being only 22-27km apart. It is noteworthy that GW locality is isolated by the River Nile and its tributaries from the other collection sites in 3 directions (North, West and East). Furthermore there are no direct routes of transportations between this and other locations in Khartoum State (see map in Figure 1), a matter that may constitute a further barrier to gene flow for the GW population. It is noteworthy that uncommon clinical manifestations of CL were also reported from SR which used to be an endemic site for common CL cases. However, *P*. *papatasi* population from this site was significantly genetically differentiated from GW than from the other two sites from Khartoum State (MO and TR). Previous studies demonstrated the presence of different genetic groups among *L*. *major* strains from different locations in the same country which could be related to different *P*. *papatasi* subpopulations [43,44]. Whether the parasite strains causing these complications in SR and GW belong to the same genetic groups or not, remains to be further explored.

It is well known that populations in close proximity are genetically more similar than distant populations and that high gene flow usually precludes local adaptation [45]. Therefore, the lack of interpopulation genetic differentiation among Khartoum State populations (MO, TR and SR) could be attributed to the flies’ own dispersal abilities, to human transportation and potentially to wind dispersal. *P. papatasi* is known to be capable of flying up to 2km per night in open desert where they can be also be carried by air currents. Moreover, sandflies usually swarm over their host during feeding and usually mating takes place on the host; thus they may be capable of movement along with animal herds [46,47]. It should be stated that the Moyleih area (MO) hosts a famous cattle market where animals are brought from Western and Southern Sudan either for slaughter in the nearby “Ghunawa” abattoir or to be herded across the Nile bridge towards the “Kadaro” abattoir on the Eastern bank of the Nile very near the village of Sirougia (SR). In addition, the seasonal movement of the camel- herding nomads northward during the wet season and southward at the end of the wet season [48] may contribute to the dispersal of sandfly over a wide range.

The close genetic similarity among sandfly populations from Khartoum State (MO, TR and SR) and Wad Shariefai (SHR), Eastern Sudan, despite the large geographical distance, could be attributed to the prevailing environmental conditions such as wind speed, humidity and temperatures that may play an important role in sandfly dispersal [1]. Although each of the studied sites has their own peculiar environmental conditions, they all lie within the dry savannah belt [49]. Moreover MO and TR, although they are apparently desert areas, used to be covered by thick savannah forests that may have had similar environmental conditions as SHR [50]. Since the mid-1970s the whole Western Sudan, including the area west of the White Nile, suffered from continuous drought periods that lead to deterioration of the plant cover, soil, water and biodiversity [50-52]. Previous studies on the distribution and phenology of *P*. *papatasi* indicate that this species can withstand extreme environmental conditions and survive even in areas with extreme temperature and aridity [53-55]. The prevailing conditions within deep wall- cracks and small muddy huts provide high humidity and cooler temperatures suitable for sandfly proliferation in these sites. All these factors may explain the genetic similarity observed between distant sandfly populations since flies can disperse largely freely between vast expenses of largely similar habitats.

Low genetic differentiation between *P*. *papatasi* populations separated by large geographical distances has also been reported in previous studies based on different molecular markers [14,56,57]. In addition, population genetic studies of other important sandfly species demonstrated the homogeneity of these populations in a radius of 20km while a certain degree of structuring was observed in a zone of approximately 1km [58]. It is worth mentioning that little genetic differentiation was also shown in populations of *P*. *orientalis* in Sudan using 30 RAPD markers. In addition, populations from Dinder National Park, South-Eastern Sudan were found to be genetically very similar to population from SR, Khartoum State [59]. It has been recently postulated that latitude is the main factor determining the degree of genetic diversity among *P. papatasi* populations [13].

This study also revealed significant genetic differentiation and restricted gene flow between the Egyptian EGY population and all the Sudanese populations, which suggests some degree of isolation-by-distance despite the overall lack of a significant correlation between the geographical distance and estimates of genetic differentiation. The Egyptian *P*. *papatasi* colony also showed unexpected high degree of heterozygosity compared to the Sudanese field populations, although long colonization and adaptation to laboratory conditions are usually thought to minimize the genetic variability in laboratory populations [60,61]. Unfortunately, no detailed information was available about the EGY colony, although it may be speculated that this diversity may be a result of its augmentation with new collections, which is a common practice in sand fly colonization. It must be mentioned that in previous studies, high levels of polymorphism and heterozygosity were also observed in *P*. *papatasi* colony from Sinai, Egypt even after the 33 generations [62].

## Conclusion

The significant differentiation of the GW *Phlebotomus papatasi* population from the other Sudanese populations may have important consequences on the epidemiology of leishmaniases in Khartoum State and warrants further investigation. Such genetic variations could potentially correlate with differences in the vectorial capacity of the sandfly vector to transmit different strains of the parasites or even hybrids. Future studies using a larger number of microsatellite loci, more populations and more specimens per population are also needed to further elucidate patterns of genetic differentiation of *P*. *papatasi* in Sudan. This knowledge would improve our understanding of the epidemiology of the disease in Sudan, and ultimately would result in improved disease and vector control programs.

## Abbreviation

CL: Cutaneous leishmaniasis; VL: Visceral leishmaniasis; MO: Moyleih, a semi-desert area on the west bank of the White Nile; TR: El-Trais, an irrigated area on the west bank of the White Nile; SR: Sirougia village, a green rich irrigated area on the east bank of the River Nile; GW: Gerif West, a residential area on the west bank of the Blue Nile; SHR: Wad Shariefai village, in Kassala State, Eastern Sudan where the three closely-related species belonging to the subgenus *Phlebotomus Phlebotomus* were found sympatrically; EGY: Colony samples from Egypt; HWE: Hardy-Weinberg equilibrium; He: Expected heterozygosity; Ho: Observed heterozygosity; SD: Standard deviation; Wright’s *F*_ST_: Measure of population differentiation; F_IS_: Inbreeding coefficient; (*Nm*): Rate of gene flow among populations.

## Competing interests

The authors declare that they have no competing interests.

## Authors’ contributions

NK carried out the field and laboratory work, microsatellite analysis and drafted the manuscript. MA, FA and DE helped in designing the field study, interpretation of the results and editing the manuscript. FT helped with the data analyses, interpretation of the results and editing the manuscript. All authors read and approved the final version of the manuscript.
